# Effect of Periodontal Instrumentation on Tooth Structure Exposure to Ionizing Radiation: *In Vitro* Study

**DOI:** 10.1590/0103-6440202405763

**Published:** 2024-10-28

**Authors:** Guilherme Gonçalves da Cruz, Roberta de Oliveira Alves, Caroline Garcia Orsi, André Luis Faria-e-Silva, Suzane Cristina Pigossi, Priscilla Barbosa Ferreira Soares

**Affiliations:** 1 Department of Periodontology and Implantology, School of Dentistry, Universidade Federal de Uberlândia, Campus Umuarama, Uberlândia, Minas Gerais, Brazil.; 2 Associate Professor. Department of Dentistry, Universidade Federal de Sergipe, Aracaju, Sergipe, Brazil

**Keywords:** radiotherapy, dental calculus, dental prophylaxis

## Abstract

This study aimed to evaluate the effects of different scaling protocols on the morphology and roughness of the root dentin substrate exposure to ionizing radiation. One hundred and thirty extracted bovine incisors were randomly divided into two groups (n=65): non-irradiated (NIR) and irradiated (IR). Each group was initially subdivided into three subgroups according to the type of non-surgical periodontal protocol: NIT: no instrumentation; HS: hand scaling with 15 apical-coronal instrument movements; US: ultrasonic scaling with 15 apical-coronal cycles. Subsequently, all samples were subjected to the prophylaxis protocol, being subdivided into the following groups: NIT/PP: prophylaxis with a fine prophylactic paste using a rubber cup for 15 seconds; HS/PP: Hand scaling followed by the prophylaxis protocol; US/PP: Ultrasonic scaling followed by the prophylaxis protocol. The roughness of the root dentin surface was measured with a profilometer (Ra/Rz - μm), and the morphology of the dentin surfaces was analyzed using scanning electron microscopy (SEM). The analyses were conducted before and after the prophylaxis protocol. In the absence of prophylaxis, the roughest surfaces were observed after ultrasonic instrumentation followed by hand instrumentation for both IR and NIR groups. No difference in Ra and RZ values between HS/PP and US/PP was observed for both substrates. For the IR group, the prophylaxis resulted in similar Ra and RZ values for both instrumentation groups in comparison to no instrumentation. Ordinal logistic regression showed that both HS and US resulted in higher scores than NIT, irrespective of IR presence. In conclusion, the IR showed a rougher root surface for both HS and US in comparison to NIR. However, the prophylaxis procedure significantly reduced the roughness of root surfaces after both instrumentation procedures

## Resumo

O objetivo deste estudo foi avaliar os efeitos de diferentes protocolos de raspagem na morfologia e rugosidade do substrato de dentina radicular exposto à radiação ionizante. Cento e trinta incisivos bovinos extraídos foram divididos aleatoriamente em dois grupos (n = 65): não irradiados (NIR) e irradiados (IR). Cada grupo foi subdividido em seis subgrupos de acordo com o tipo de protocolo periodontal não cirúrgico: NIT: sem instrumentação; NIT/PP: profilaxia com pasta profilática fina usando uma taça de borracha por 15 segundos; HS: raspagem manual com 15 movimentos de instrumento apical-coronal; HS/PP: raspagem manual seguida de protocolo de profilaxia; US: raspagem ultrassônica com 15 ciclos apical-coronais; US/PP: raspagem ultrassônica seguida de protocolo de profilaxia. A rugosidade da superfície da dentina radicular foi medida com um perfilômetro (Ra/Rz - μm) e a morfologia das superfícies da dentina foi analisada usando microscopia eletrônica de varredura (SEM). Na ausência de profilaxia, as superfícies mais ásperas foram observadas após a instrumentação ultrassônica seguida de instrumentação manual para ambos os grupos IR e NIR. Não foi observada diferença nos valores de Ra e RZ entre HS/PP e US/PP para ambos os substratos. Para o grupo IR, a profilaxia resultou em valores de Ra e RZ semelhantes para ambos os grupos de instrumentação em comparação com a ausência de instrumentação. A regressão logística ordinal mostrou que tanto HS quanto US resultaram em escores mais altos do que NIT, independentemente da presença de IR. Em conclusão, o IR apresentou uma superfície radicular mais áspera para ambos os HS e US em comparação com o NIR. No entanto, o procedimento de profilaxia reduziu significativamente a rugosidade das superfícies radiculares após ambos os procedimentos de instrumentação.

## Introduction

Head and neck cancer (HNC) represents one of the most common malignant neoplasms, with exponential growth in cases ^1^. Radiotherapy (RT) plays a key role in HNC treatment, being indicated as a unique therapy in early-stage tumors or combined with surgery or chemotherapy in advanced stages ^2^. However, RT can adversely affect the oral cavity tissues when applied to the head and neck region since the ionizing radiation has cytotoxic effects against normal and malignant cells ^3^. Consequently, the HNC radiotherapy treatment has been associated with diverse oral sequelae such as mucositis, hyposalivation, radiation dermatitis, oropharyngeal candidiasis, mucosal pain, taste dysfunction, trismus, temporomandibular dysfunction, dental caries, periodontitis, and osteoradionecrosis ^4^ ^,^ ^5^.

In addition, RT also causes damage to the tooth structure, which affects the mechanical properties and chemical composition of enamel (crystalline structure, acid solubility, and microhardness) and dentin (elastic modulus, microhardness, matrix metalloproteinases releasing) ^6^ ^,^ ^7^ ^,^ ^8^. These changes associated with xerostomia caused by hyposalivation can lead to rapid and severe destruction of enamel and dentin ^9^. Moreover, oral hygiene maintenance can be difficult by RT due to painful sensations associated with xerostomia in the mucosa ^10^. All these changes can impair the patients to adequately control the oral biofilm increasing the risk of periodontal attachment loss and caries ^5^.

The calculus and biofilm removal carried out during non-surgical periodontal therapy is essential to control periodontal infection and achieve satisfactory periodontal health in HNC patients submitted to RT ^5^. This therapy can be made using manual (periodontal curettes) or ultrasonic (ultrasound) instrumentation ^11^. However, these instruments can increase the root surface roughness by increasing the irregularities and sulcus, resulting in greater biofilm adhesion ^12^ ^,^ ^13^. Therefore, surface polishing (called prophylaxis) should be made to decrease the residual root roughness after periodontal instrumentation ^14^.

Based on that, this study evaluated the effect of different instrumentation methods (hand or ultrasonic instrumentation, followed or not by prophylaxis) on the root dentin surface roughness of irradiated and non-irradiated bovine teeth. The null hypothesis tested was that both instrumentation methods and tooth irradiation do not affect the roughness of root dentin.

## Materials and methods

### Sample separation and irradiation

One hundred and thirty extracted bovine incisors were selected for this study. The teeth were stored at 4ºC in distilled water that was replaced weekly. The crowns were separated from the roots at the enamel-cementum junction with a double-faced diamond disk (KG Sorensen, Barueri, SP, Brazil) used with a low-speed handpiece (KaVo do Brasil Ltda, Joinville, SC, Brazil) under copious water spray. A 5 x 5 mm² section was delimited on the buccal root surface and fixed within a rectangular block of polystyrene resin (Cristal, Piracicaba, SP, Brazil). The block measured 2.5 cm in length, 1.5 cm in width, and 1.0 cm in height was obtained with a polyether impression material (Impregum Soft; 3M ESPE, St. Paul, MN, USA) matrix. The surface to be analyzed was flattened using 600-, 800-, 1200- and 2000-grit silicon-carbide papers (Norton, Campinas, SP, Brazil) and polished with metallographic diamond pastes (6, 3, 1, 1⁄4 μm; Arotec, São Paulo, SP, Brazil). The samples were cleaned with three 10-minute ultrasound baths (Cristofoli, Campo Mourão, Paraná, Brazil) with absolute alcohol to remove debris.

**Figure 1:**
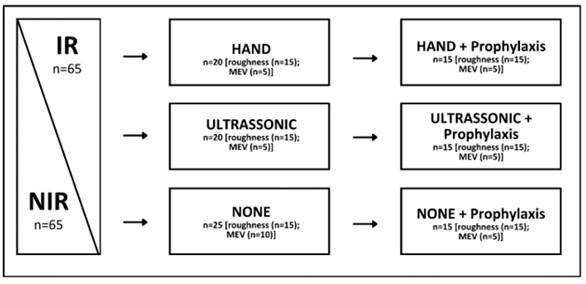
Study design

The samples were randomly divided into two groups (n=65): non-irradiated (NIR) and irradiated (IR) (Figure 1). The IR samples were fixed in utility wax plates (Technew, Rio de Janeiro, RJ, Brazil) and completely immersed in distilled water at room temperature during irradiation. The distilled water was discarded every 15 days, and the specimens were stored in new distilled water. The irradiation protocol consisted of a total dose of 72 Gy, with 1,8 Gy daily applied five days a week, for eight weeks with X-rays from a linear accelerator (Clinac 600C Varian®-Palo Alto, CA, USA, Beam 6 MV). The NIR samples were stored in distilled water at 4^◦^C during the time required to complete the irradiation protocol ^6^.

### Instrumentation

After one week following the completion of the radiotherapy protocol both IR and NIR samples were randomly divided to receive one of the following instrumentations (Figure 1): Hand (n=20) - scaling using hand instrumentation (Gracey curettes 5/6; Hu-Friedy, Chicago, Illinois, USA) with 15 apical-coronal movements; Ultrasonic (n=20): scaling using ultrasonic instrumentation (Piezon PM200; EMS, Nyon, Switzerland), with 15 apical-coronal cycles; or no-instrumentation (n=25). The samples were fixed on a bench vise (185089, MTX, China) during the instrumentation/prophylaxis procedures. One operator performed all scaling and polishing procedures. A second blinded operator evaluated the samples with a profilometer (Surftest SJ-201P; Mitutoyo Corporation, Kawasaki, Kanagawa, Japan).

### Determination of the Root Surface Roughness

The surface roughness was measured before and after the interventions (n=15 samples in each group). The root surface roughness was measured with a profilometer. Five parallel readings were performed on an area previously delimited, and the average was calculated. The readings were performed with a 0.25-mm cut-off and 1.25-mm measurement length at a speed of 1 mm/s, covering 3 mm. It was calculated both the mean of the recorded peaks and valleys (Ra) and mean roughness depth (Rz), which is the maximum distance between the highest peak and the deepest valley. The profilometer was positioned in a manner that the height of the reading tip was adapted to the previous map of each sample.

### Post-instrumentation prophylaxis

After the measurement of surface roughness, all samples (n=15 in each group) were submitted to prophylaxis with a fine prophylactic paste (Herjos-F; Vigodent S.A., Rio de Janeiro, RJ, Brazil). A rubber cup coupled to a low-speed rotation device was used under constant irrigation for 15 seconds. The surface roughness was measured again, as described previously.

### Scanning Electronic Microscopy (SEM) Analysis

The morphological features of the root surfaces were analyzed using SEM. Five samples from each experimental condition were randomly selected for this analysis. For the control group (no instrumentation and no prophylaxis), ten samples from NI and NIR groups were analyzed. These samples were cleaned in an ultrasonic bath (Cristofoli, Campo Mourão, Paraná, Brazil) with distilled water for 30 minutes to remove the debris, followed by dehydration in ascendant concentrations of ethanol (50º, 70º, and 95º) for 10 min in each. Then samples were placed into absolute ethanol for 30 min. After storage in an oven receptacle containing silica for eight hours to remove moisture, the specimens were mounted on an aluminum stub (one stub per group), sputter-coated with a thin layer of gold, and examined with a VEGA 3 LMU scanning electron microscope (TESCAN, Libušina, Czech Republic). The SEM photomicrographs at ×500 magnification were scored individually by five blinded investigators based on the roughness and loss of tooth substance index based on Meyer & Lie, 1977 ^15^: [0] - smooth and even root surface without marks from instrumentation and with no loss of tooth substance; [1] - slightly roughened as corrugated local areas confined to the cementum; [2] - definitely corrugated local areas where the cementum may be completely removed, although most of the cementum is still present; [3] - considerable loss of tooth substance with instrumentation marks into the dentin. The cementum is completely removed in large areas or has many lesions from the instrumentation. This index was used to qualitatively score the morphology of the root surface produced by each instrument with or without prophylaxis.

### Data analysis

Normal distribution of final values of Ra and Rz were analyzed using the Shapiro-Wilk and Levene test. The assumption of sphericity of checked using Mauchly's W and the data were submitted to Repeated-measures ANOVA. Tukey`s test was used for multiple comparisons. Regarding the scores attributed to SEM images, agreement among the evaluators was assessed by Fleiss Kappa. The score of each specimen was defined as the mode (most observed value) calculated from the scores attributed by the evaluators. Ordinal logistic regression was used to analyze the effect of each factor on scores. A significance level of ( = 0.05 was used for all data analyses.

## Results

### Evaluation of root surface roughness - Ra

A repeated-measures ANOVA revealed that “substrate” (P < .001), “instrumentation” (p < .001), and “post-instrumentation prophylaxis” (P < 0.001) affected the final Ra values. P-values calculated for all double interactions and the triple interaction were also significant (P < 0.001 for all). In the absence of prophylaxis, the roughest surfaces were observed after ultrasonic instrumentation, followed by hand instrumentation for both IR and NIR groups. No difference in Ra values between hand and ultrasonic instrumentation was observed after prophylaxis for both substrates. For the IR group, the prophylaxis resulted in similar Ra values for both instrumentation groups in comparison to no instrumentation. The substrate irradiation affected the Ra values only when the instrumentation (hand or ultrasonic) was not followed by the prophylaxis (Table 1).

**Table 1:** Means (standard deviation) of final Ra values (in μm) according to substrate, instrumentation, and post-instrumentation prophylaxis (n = 15).

Substrate		Non-irradiated		Irradiated	
Post-instrumentation prophylaxis		Without	With	Without	With
Instrumentation	Hand	0.31 (0.05) Ba	0.22 (0.04) Aa	0.46 (0.07) Ba*	0.13 (0.04) Ab
	Ultrasonic	1.51 (0.23) Aa	0.25 (0.06) Ab	2.32 (0.25) Aa*	0.20 (0.03) Ab
	None	0.06 (0.01) Ca	0.11 (0.02) Ba	0.05 (0.01) Ca	0.10 (0.02) Aa

For each irradiation, distinct letters (uppercase comparing Instrumentation; lowercase comparing post-instrumentation prophylaxis) indicate statistical difference at Tuckey`s test (p < .05). * Indicates statistical difference from non-irradiated substrate for a same instrumentation and post-instrumentation prophylaxis at Tuckey`s test (p < .05).

### Evaluation of root surface roughness - Rz

A repeated-measures ANOVA showed that the factors “instrumentation” (P < .001), “substrate” (P < 0.001), and “post-instrumentation prophylaxis” (P < 0.001) affected the final values of Rz. All double interactions and the triple interaction had significant p-values (P < 0.001 for all). In the absence of prophylaxis, the highest values of Rz were observed for ultrasound instrumentation, followed by hand instrumentation, in both IR and NIR groups. After prophylaxis, a reduction in Rz values was observed only for ultrasonic instrumentation in the NIR group. In the IR group, a smoother surface was obtained with prophylaxis after both ultrasonic and hand instrumentation. After prophylaxis, no difference in roughness was observed among hand, ultrasonic, and no instrumentation (Table 2). Irradiating the substrate affected the roughness (rougher surface) only when hand or ultrasonic instrumentations were not followed by prophylaxis.

**Table 2:** Means (standard deviation) of final Rz values (in μm) according to substrate, instrumentation, and post-instrumentation prophylaxis (n = 15).

Substrate		Non-irradiated		Irradiated	
Post-instrumentation prophylaxis		Without	With	Without	With
Instrumentation	Hand	1.67 (0.23) Ba	1.10 (0.22) Aa	2.53 (0.48) Ba*	0.69 (0.21) Ab
	Ultrasonic	6.64 (0.87) Aa	0.97 (0.20) Ab	9.63 (1.62) Aa*	1.03 (0.22) Ab
	None	0.34 (0.08) Ca	0.63 (0.09) Aa	0.32 (0.06) Ca	0.57 (0.13) Aa

For each irradiation, distinct letters (uppercase comparing instrumentation; lowercase comparing post-instrumentation prophylaxis) indicate statistical difference at Tuckey`s test (p < .05). * Indicates statistical difference from non-irradiated substrate for a same instrumentation and post-instrumentation prophylaxis at Tuckey`s test (p < .05).

**Table 3:** Model coefficients to estimate the scores calculated using the Ordinal logistic regression.

Predictor	Estimate	SE	p-value
Substrate:			
Irradiated vs. Non-irradiated (ref.)	0.031	0.559	0.956
Instrumentation:			
Hand vs. None (ref.)	2.477	0.802	0.002
Ultrasonic vs. None (ref.)	6.221	1.149	< 0.001
Post-instrumentation prophylaxis:			
With vs. Without (ref.)	-3.085	0.713	< 0.001
			

SE: Standard error.

### Scores (SEM images)

**Figure 2:**
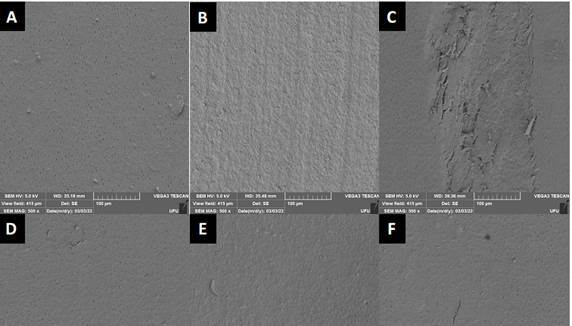
SEM images (500 x) showing the morphology of root surfaces of different interventions of No Irradiated group: (A) No intervention; (B) Scaling with hand instrumentation; (C) Scaling using a ultrasonic instrumentation; (D) Prophylaxis; (E) Scaling with hand instrumentation followed by prophylaxis; (F) Scaling using a ultrasonic instrumentation followed by prophylaxis.

**Figure 3:**
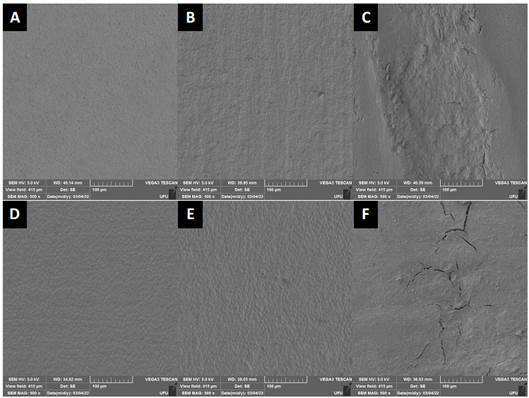
SEM images (500 x) showing the morphology of root surfaces of different interventions of Irradiated group: (A) No intervention; (B) Scaling with hand instrumentation; (C) Scaling using a ultrasonic instrumentation; (D) Prophylaxis; (E) Scaling with hand instrumentation followed by prophylaxis; (F) Scaling using a ultrasonic instrumentation followed by prophylaxis.

The repeated-measures ANOVA revealed a moderate concordance among the evaluators was observed (overall Kappa = 0.443). The highest agreement was observed for the score 3 (Kappa = 0.872) and the lowest for the score 1 (Kappa = 0.155). The results of Ordinal logistic regression are presented in Table 3. In general, the substrate did not affect the scores (Figures 2A and 3A). On the other hand, both manual and ultrasonic instrumentation resulted in higher scores than no instrumentation (Figures 2B, 2C, 3B, and 3C), and the scores were reduced after prophylaxis (Figure 2E, 2F, 3E, and 3F).

## Discussion

To the author's best knowledge, it is noteworthy this is the first in vitro study that evaluated the effect of different scaling protocols on the morphology and roughness of the root dentin substrate exposure to ionizing radiation. Our findings demonstrated that the most pronounced surface alterations were observed in irradiated surfaces after the ultrasonic instrumentation, rejecting the null hypothesis. In addition, no difference in roughness was observed among hand, ultrasonic, and no instrumentation after post-instrumentation prophylaxis in IR and NIR samples. This last finding suggests that prophylaxis can overcome the tooth surface alterations caused by the instrumentation during periodontal treatment.

Different methods have been proposed to analyze changes in the root surface after applying periodontal instruments, including SEM, atomic force microscope, histological evaluation, three-dimensional optical laser scanner, profilometer, and computerized tomography. It is important to emphasize that each technique has particularities and limitations ^12^ ^,^ ^13^ ^,^ ^16^ ^,^ ^17^. The present study measured the surface roughness using a contact profilometer device, while surface wear was identified using an SEM. Profilometer analysis is a two-dimensional measurement that provides reliable data for characterizing root surface roughness after debridement, and it is one of the common methods used for surface roughness analysis ^16^ ^,^ ^18^ ^,^ ^19^. The results of the analysis of roughness were associated with the root wear data observed through SEM images, seeking to improve the characterization of morphological features on the root surfaces. A moderate agreement between the evaluators determining the SEM scores was observed, and the highest agreement occurred for score 3, which indicates more extensive surface wear.

Regarding the roughness parameters after instrumentation, Ra and Rz values showed that ultrasound instrumentation yields the roughest root surfaces, irrespective of the irradiation of the tooth substrate. However, for both instrumentation methods, the surface roughness increase was more pronounced for irradiated substrates. These results may be related to the possible changes in both organic and inorganic contents caused by the ionizing radiation on the dental substrate. Collagen is the most abundant protein in dentin (≈ 90%), and its proteolysis significantly impacts the tissue's structural integrity ^6^. Some studies evaluating changes in the chemical composition of the dental structure indicate a drop in the amide I/amide III and amide I/CH_2_ ratios. These ratio reductions negatively affect the quality and organization of collagen fibrils and ultimately compromise the physical and mechanical properties of the dentin ^6^ ^,^ ^20^. As a consequence of that, a more pronounced instrumentation effect on surface roughness can be expected in substrates with poorer mechanical properties ^6^ ^,^ ^20^ ^,^ ^21^ ^,^ ^22^.

The polishing of the root surfaces exposed to the oral environment is indicated to reduce the biofilm adhesion, strengthening and facilitating the biofilm mechanical control by the patient ^16^. In the present study, the prophylaxis was able to smooth the rough surfaces (Ra and Rz) observed after hand and ultrasonic instrumentations. This reduction in roughness parameters after prophylaxis corroborates with other studies, and it supports the importance of carrying out this procedure during non-surgical periodontal treatment in patients more susceptible to periodontal disease submitted to RT in the head and neck region ^4^ ^,^ ^16^ ^,^ ^23^. It is also important to report that the prophylaxis procedure in the IR group was more easily executed, which can be demonstrated by lower Ra and Rz values for this group. This observation suggested that reducing phosphate and carbonate concentrations also reduces the dentin microhardness and elastic modulus ^6^ ^,^ ^20^ ^,^ ^21^ ^,^ ^22^. In addition, prophylaxis alone did not significantly change the surface roughness in the present study.

The in vitro model developed in the present study simulated the RT for HNC cancer treatment with a rigorous experimental variables control. However, data extrapolation to clinical practice should be done carefully. Several in vitro studies have used human-extracted teeth as substrate. However, further to ethical restrictions, the standardization of samples obtained from bovine incisor teeth is simplest compared to human teeth (e.g., source, age, and other). In addition, similar physical and mechanical properties were observed between bovine and human teeth ^21^ ^,^ ^22^ ^,^ ^24^ ^,^ ^25^. Regarding the storage solutions, physiological saline solution, artificial saliva, or distilled water have been used ^8^ ^,^ ^21^. As the focus of this study was the direct effect of scaling on irradiated dental tissue, distilled water was chosen as the storage solution. Distilled water provides an environment capable of radiolysis without greater interaction with the teeth.

## Conclusion

In conclusion, irradiated dentin showed a rougher root surface for both manual and ultrasonic instrumentation compared to non-radiated dentin. However, the prophylaxis procedure significantly reduced the roughness of root surfaces after both instrumentation procedures.
